# GSK1059615 kills head and neck squamous cell carcinoma cells possibly via activating mitochondrial programmed necrosis pathway

**DOI:** 10.18632/oncotarget.15135

**Published:** 2017-02-07

**Authors:** Jing Xie, Quan Li, Xi Ding, Yunyun Gao

**Affiliations:** ^1^ Department of Stomatology, The First Affiliated Hospital of Wenzhou Medical University, Wenzhou, China; ^2^ Center of Stomatology, The Second Affiliated Hospital of Soochow University, Suzhou, China

**Keywords:** HNSCC, PI3K-AKT-mTOR, GSK1059615, programmed necrosis

## Abstract

This study tested the anti-head and neck squamous cell carcinoma (HNSCC) cell activity by GSK1059615, a novel PI3K and mTOR dual inhibitor. GSK1059615 inhibited survival and proliferation of established (SCC-9, SQ20B and A253 lines) and primary human HNSCC cells. GSK1059615 blocked PI3K-AKT-mTOR activation in HNSCC cells. Intriguingly, GSK1059615 treatment in HNSCC cells failed to provoke apoptosis, but induced programmed necrosis. The latter was tested by mitochondria depolarization, ANT-1-cyclophilin-D mitochondrial association and lactate dehydrogenase (LDH) release. Reversely, mPTP blockers (sanglifehrin A, cyclosporin A and bongkrekic acid) or cyclophilin-D shRNA dramatically alleviated GSK1059615-induced SCC-9 cell death. Further studies demonstrated that GSK1059615 *i.p*. injection suppressed SCC-9 tumor growth in nude mice, which was compromised with co-administration with cyclosporin A. Thus, targeting PI3K-AKT-mTOR pathway by GSK1059615 possibly provokes programmed necrosis pathway to kill HNSCC cells.

## INTRODUCTION

Head and neck squamous cell carcinoma (HNSCC) is a large heterogeneous family of carcinomas, including carcinomas of face, nasopharynx, oral cavity, and larynx [1–3]. The overall survival is still poor for those with high-degree or malignant tumors [1–4]. One major signaling that is often dysregulated in HNSCC is phosphatidylinositol 3-kinase-AKT-mammalian target of rapamycin (PI3K-AKT-mTOR) cascade [5–7]. Recent research efforts have developed a novel PI3K and mTOR dual inhibitor, named GSK1059615 [8]. As compared to other PI3K-AKT-mTOR specific inhibitors, the advantage of this compound is significant, as it simantanuously blocks PI3K and mTOR [8]. Therefore, it has the potential to completely shut down the PI3K-AKT-mTOR cascade [8]. The potential effect of GSK1059615 on HNSCC cells is evaluated in this preclinical study.

Recent studies have proposed a non-apoptotic form of cell death named programmed necrosis, which also requires mitochondrial permeability transition pore (mPTP) opening [9–13]. The channel complex mPTP has several major components, including voltage-dependent anion channel (VDAC, in outer membrane), the adenine nucleotide translocator-1 (ANT-1, in the inner membrane), cyclophilin-D (in the matrix) [14] [14–16]. Many cancer-killing agents are capable of inducing mitochondrial depolarization and ANT-1-cyclophilin-D association, causing mPTP opening and subsequent cell necrosis (but not apoptosis) [9–13]. In the present study, we show that GSK1059615 also provokes programmed necrosis pathway in HNSCC cells.

## RESULTS

### GSK1059615 is cytotoxic to both established and primary human HNSCC cells

**Figure 1:**
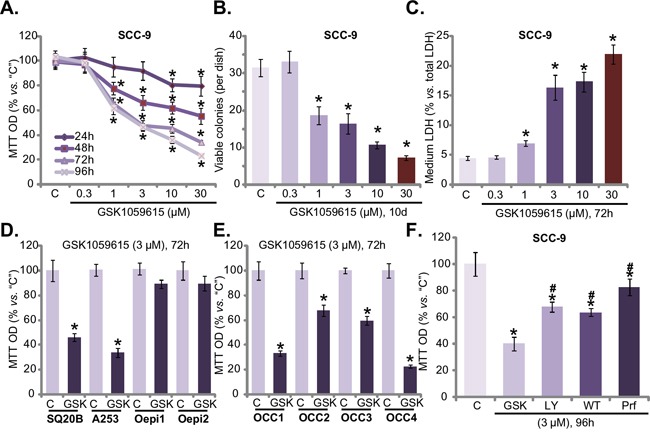
GSK1059615 is cytotoxic to HNSCC cells HNSCC cell lines (SCC-9, SQ20B and A253) **A-D**, **F.**, primary human OCC cells (“OCC1-4”) **E.** or oral epithelial cell (“Oepi1/2”) (D) were treated with designated concentration of GSK1059615 (“GSK”), or wortmannin (“WT”), LY294002 (“LY”) and perifosine (“Prf”), cells were further cultured for indicated time period, and cell survival was tested by listed assays. “C” stands for untreated control group (Same for all Figures). For each assay, n=6. Bars stand for mean ± SD (Same for all Figures). Experiments in this figure were repeated four times, and similar results were obtained. * *p* < 0.01 vs. group “C”.

First, we tested the potential effect of GSK1059615 on HNSCC cell survival. SCC-9 cells, cultured in complete medium (with 10% FBS), were treated with gradually increasing concentration (0.3-30 μM) of GSK1059615, and tetrazolium dye (MTT) assay was applied to test cell survival. Results in Figure 1A demonstrated that treatment with GSK1059615 in SCC-9 cells dose-dependently inhibited cell survival. The MTT OD was significantly decreased following GSK1059615 (1-30 μM) treatment (Figure 1A). Notably, it took at least 48h for the GSK1059615 treatment to exert significant anti-survival activity (Figure 1A). The IC50 of GSK1059615, or the concentration that inhibited 50% of cell survival, was close to 3 μM (72h and 96h treatment duration) (Figure 1A). To further confirm the anti-survival activity by GSK1059615, clonogenicity assay was performed. Results in Figure 1B showed that GSK1059615, at 1-30 μM, dramatically decreased the number of viable SCC-9 colonies. The lactate dehydrogenase (LDH) content was also significantly increased in the conditional medium of GSK1059615 (1-30 μM)-treated cells (Figure 1C), implying that GSK1059615 induced SCC-9 cell death.

The activity of GSK1059615 on other HNSCC cells was also tested. In both A253 and SQ20B HNSCC cells [17], GSK1059615 (3 μM, 72h) largely decreased cell survival (MTT OD, Figure 1D). On the other hand, the very same GSK1059615 treatment failed to inhibit the survival of two oral epithelial cell lines (“Oepi1/2”) (Figure 1D), implying that GSK1059615 could be cytotoxic only to cancer cells. In order to test the effect of GSK1059615 in primary cancer cells, a total of four lines of primary (“patient-derived”) oral cavity carcinoma (OCC) cells were established (named “OCC1-4”), which were also treated with GSK1059615 (3 μM, 72h). MTT assay results in Figure 1E showed that GSK1059615 was cytotoxic to all the primary cancer cells. Remarkably, we found that GSK1059615 was more potent that other known AKT inhibitors (i.e. LY294002, Wortmannin and perifosine) in killing SCC-9 cells (Figure 1F). Together, these results demonstrate that GSK1059615 is cytotoxic to established and primary human HNSCC cells.

### GSK1059615 inhibits human HNSCC cell proliferation

**Figure 2:**
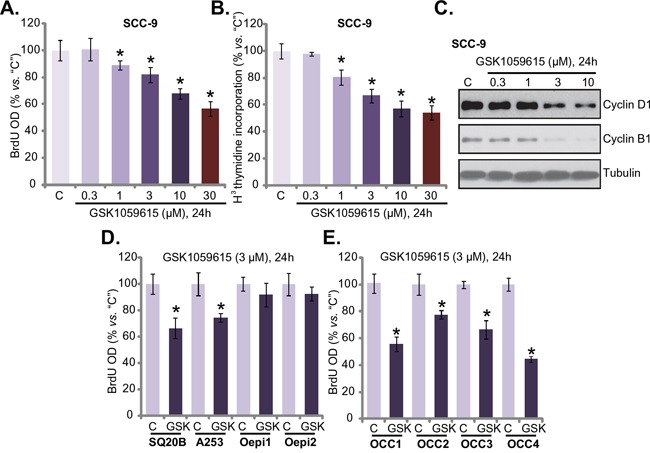
GSK1059615 inhibits HNSCC cell proliferation HNSCC cell lines (SCC-9, SQ20B and A253) **A-D.**, primary human OCC cells (“OCC1-4”) **E.** or oral epithelial cell (“Oepi1/2”) (D) were treated with designated concentration of GSK1059615 (“GSK”), cells were further cultured for indicated time period, cell proliferation was tested by BrdU ELISA assay (A, D and E) and [H^3^] thymidine incorporation assay (B); Expression of proliferation-associated proteins was tested by Western blot assay (C) For each assay, n=5. Experiments in this figure were repeated three times, and similar results were obtained. * *p* < 0.01 vs. group “C”.

Cytotoxicity in HNSCC cells could be due to proliferation inhibition. Next, proliferation of GSK1059615-treated HNSCC cells was tested by the BrdU ELISA assay and [H^3^] thymidine incorporation assay [18]. Results from both assays demonstrated clearly that GSK1059615 dose-dependently inhibited SCC-9 cell proliferation (Figure 2A and 2B), as the BrdU ELISA OD (Figure 2A) and [H^3^] thymidine incorporation (Figure 2B) were both decreased following GSK1059615 (1-30 μM) treatment. Expression of proliferation-associated proteins, including cyclin D1 and cyclin B1, was also significantly downregulated following GSK1059615 (1-10 μM) treatment (Figure 2C). Notably, to test cell proliferation, cells were incubated with GSK1059615 for only 24h, when no significant cytotoxicity was yet noticed (Figure 1A).

BrdU ELISA assay was also performed to test proliferation of other HNSCC cells with GSK1059615 treatment. Results in Figure 2D showed clearly that GSK1059615 (3 μM) was anti-proliferative in two other HNSCC cell lines: SQ20B and A253. Yet, the same GSK1059615 treatment failed to inhibit proliferation of oral epithelial cells (“Oepi1/2”) (Figure 2D). In the primary OCC cells (all four lines, “OCC1-4”), treatment with GSK1059615 (3 μM, 24h) also inhibited cell proliferation, which was again indicated by BrdU ELISA OD reduction (Figure 2E). Collectively, these results imply that GSK1059615 inhibits human HNSCC cell proliferation.

### GSK1059615 blocks PI3K-AKT-mTOR activation in HNSCC cells

**Figure 3:**
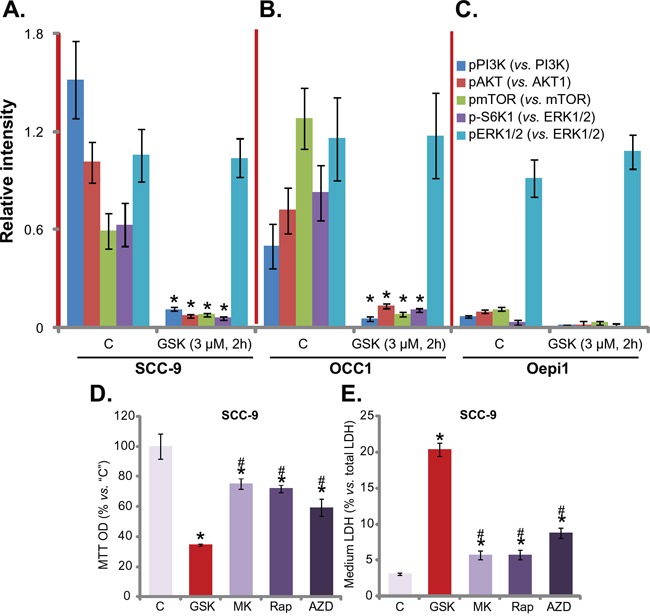
GSK1059615 blocks PI3K-AKT-mTOR activation in HNSCC cells SCC-9 cells **A.**, primary human OCC cells (“OCC1”) **B.** or oral epithelial cells (“Oepi1”) **C.** were treated with GSK1059615 (“GSK”, 3 μM) for 2h, expression of listed kinase proteins in the fresh cell lysates was tested, and data were quantified (three repeats). SCC-9 cells were treated with 3 μM of MK-2206 (“MK”), rapamycin (“Rap”) or AZD-2014 (“AZD”) for 72h, cell viability (MTT assay, **D.**) and cell death (LDH assay, **E.**) were tested. Experiments in this figure were repeated three times, and similar results were obtained. * *p* < 0.01 vs. group “C”. ^#^ *p* < 0.01 vs. GSK1059615 only (D and E).

GSK1059615 is a potent PI3K-mTOR duel inhibitor, we thus tested PI3K-AKT-mTOR signaling in GSK1059615-treated cells. The quantified results in Figure 3A and 3B showed that, treatment with GSK1059615 (3 μM) in SCC-9 cells and “OCC1” primary cancer cells dramatically inhibited phosphorylation (“p-”) of PI3K p85 (Tyr-458), AKT (Ser-473), mTOR (Ser-2448) and S6K1 (Thr-389). Thus, GSK1059615 apparently blocked PI3K-AKT-mTOR signaling cascade activation in HNSCC cells (Figure 3A and 3B). Remarkably, the basal activation of PI3K-AKT-mTOR cascade was quite low in the oral epithelial cells (“Oepi1”) (Figure 3C). p-PI3K p85, p-AKT, p-mTOR and p-S6K1 were almost undetected in the epithelial cells (Figure 3C). These might explain why these epithelial cells were not killed by GSK1059615 (Figure 1). Interestingly, ERK activation, tested by p-ERK1/2 (Thr-202/Tyr-204), was not altered by the same GSK1059615 treatment (Figure 3A-3C). Thus, GSK1059615 blocks PI3K-AKT-mTOR activation in HNSCC cells.

Next, we compared the activity of GSK1059615 with other PI3K-AKT-mTOR specific inhibitors. Results in Figure 3D and 3E showed that GSK1059615 was significantly more potent in killing SCC-9 cells than same concentration (3 μM) of the AKT specific inhibitor MK-2206 [19, 20], mTORC1 inhibitor rapamycin [21] and mTOR kinase inhibitor AZD-2014 [17, 22] (Figure 3D and 3E). GSK1059615 resulted in more survival loss (Figure 3D) and cell death (Figure 3E) than the above specific inhibitors.

### GSK1059615 fails to provoke apoptosis in HNSCC cells

**Figure 4:**
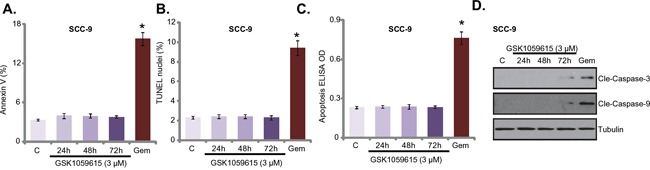
GSK1059615 fails to provoke apoptosis in HNSCC cells SCC-9 cells were treated with designated GSK1059615 (“GSK”) or gemcitabine (“Gem”, 500 nM, 48h), cells were further cultured and cell apoptosis was tested by the listed assays **A-C.** Expression of caspase proteins was examined by Western blot assay **D.** For each assay, n=5. Experiments in this figure were repeated three times, and similar results were obtained. * *p* < 0.01 vs. group “C”.

We also tested the potential effect of GSK1059615 on cell apoptosis. Three different assays were performed, including the Annexin V FACS assay, Terminal deoxynucleotidyl transferase dUTP nick end labeling (TUNEL) staining assay and Histone DNA apoptosis ELISA assay. To our surprise, the results of these assays showed that GSK1059615 (3 μM, the cytotoxic dose) failed to induce significant apoptosis in SCC-9 cells (Figure 4A-4C). Notably, to test cell apoptosis, cells were treated with GSK1059615 for three different time points (24h/48h/72h), yet no apoptosis activation was noticed at any time point (Figure 4A-4C). On the other hand, gemcitabine, served as a positive control, provoked profound apoptosis in SCC-9 cells (Figure 4A-4C). Further studies showed that gemcitabine, but not GSK1059615, induced obvious caspase-3/caspase-9 cleavage in SCC-9 cells (Figure 4D). Together, GSK1059615, although is cytotoxic, but fails to induce HNSCC cell apoptosis.

### GSK1059615 provokes programmed necrosis in HSNCC cells

**Figure 5:**
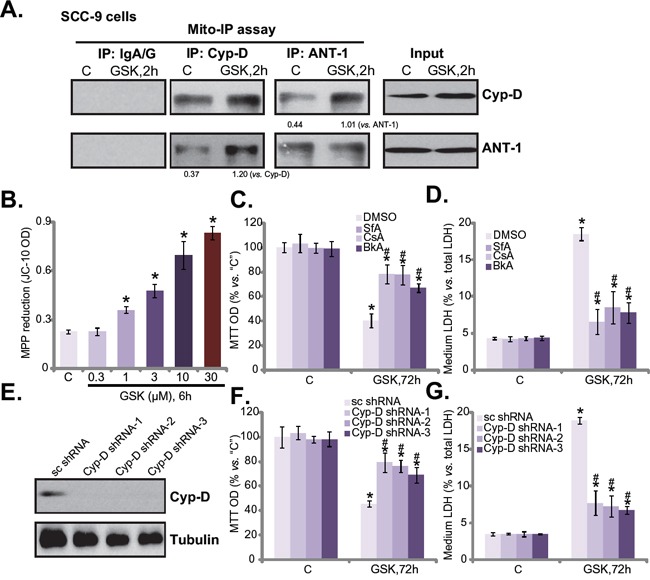
GSK1059615 provokes programmed necrosis in HSNCC cells SCC-9 cells were treated with GSK1059615 (“GSK”, 3 μM) for applied time, mitochondrial lysates were isolated, cyclophilin-D and ANT-1 association was tested by mitochondrial co-immunoprecipitation (“mito-IP”) assay (**A.**, data were quantified); Expression of cyclophilin-D (“Cyp-D”) and ANT-1 in mitochondrial lysates was also tested as “Input” (A); JC-10 intensity assay was performed to reflect mitochondrial depolarization **B.** SCC-9 cells, pre-treated for 1h with 1.0 μM of sanglifehrin A (“SfA”), cyclosporin A (“CsA”) or bongkrekic acid (“BkA”), followed by GSK1059615 (“GSK”, 3 μM) treatment for 72h, MTT cell viability **C.** and LDH release **D.** were tested. SCC-9 cells expressing cyclophilin-D shRNAs (“Cyp-D shRNA1/2/3”), or scramble control shRNA (“sc-shRNA”), were treated with/out GSK1059615 (“GSK”, 3 μM, 72h), cyclophilin-D expression **E.**, MTT cell viability **F.** and LDH release **G.** were tested. For each assay, n=5. Experiments in this figure were repeated three times, and similar results were obtained. * *p* < 0.01 vs. group “C”. ^#^ *p* < 0.01 vs. GSK1059615 only (C and D). ^#^ *p* < 0.01 vs. “sc-shRNA” (F and G).

Recent studies have shown that several anti-cancer agents could provoke mitochondrial programmed necrosis pathway to kill cancer cells [9, 12, 23–25]. Our results here also indicated activation of this necrosis pathway by GSK1059615. Mitochondrial co-immunoprecipitation assay (“mito-IP”) assay results in Figure 5A showed that GSK1059615 (3 μM) in SCC-9 cells induced cyclophilin-D-adenine nucleotide translocator-1 (ANT-1) association in the mitochondria, which is the initial step of programmed necrosis pathway activation [9, 12, 23–25]. Further, GSK1059615 dose-dependently induced mitochondrial membrane potential (MMP) collapse or mPTP opening, which was evidenced by JC-10 fluorescence intensity increase [9, 12, 23–25] (Figure 5B). Remarkably, several mPTP inhibitors, including cyclosporin A [26, 27], sanglifehrin A [26], and bongkrekic acid [28], significantly attenuated GSK1059615-induced SCC-9 cell viability reduction (Figure 5C) and cell death (Figure 5D).

The above results suggest that GSK1059615 provokes programmed necrosis to kill SCC-9 cells. To further this hypothesis, shRNA method was applied to stably knockdown cyclophilin-D, the key component of mPTP. A total of three non-overlapping cyclophilin-D shRNAs (from Dr. Xu [29]) were applied: cyclophilin-D shRNA-1/2/3. All three of them efficiently downregulated cyclophilin-D in SCC-9 cells (Figure 5E). Remarkably, GSK1059615 (3 μM)-induced cytotoxicity was largely alleviated in the cyclophilin-D-silenced cells (Figure 5F and 5G). Thus, in line with the above pharmacological evidences, the genetic evidences here further confirmed that programmed necrosis pathway mediates GSK1059615-induced cytotoxicity against SCC-9 cells.

### GSK1059615 inhibits SCC-9 tumor growth in nude mice, and its activity is compromised with cyclosporin A co-administration

**Figure 6:**
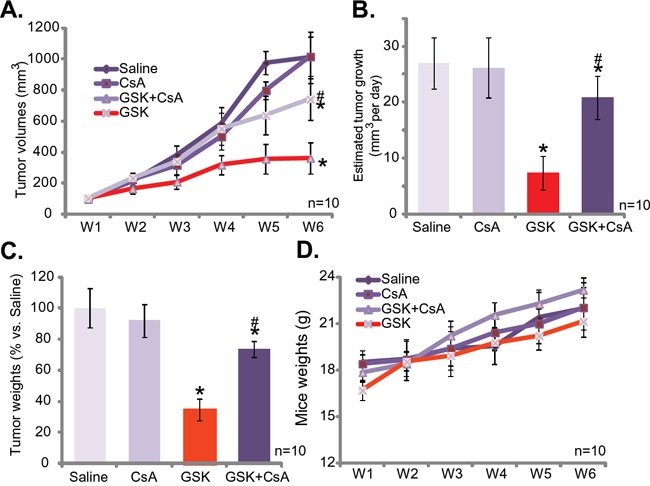
GSK1059615 inhibits SCC-9 tumor growth in nude mice SCC-9 tumor-bearing nude mice were administrated with GSK1059615 (“GSK”, 30 mg/kg, *i.p*., daily) and/or cyclosporin A (“CsA”, 5 mg/kg, *i.v*., daily), tumor volumes **A.** and mice body weights **D.** were recorded every week for a total of 5 weeks; Estimated daily tumor growth was also calculated **B.**; At the end of experiment, SCC-9 tumors were isolated and weighted **C.** For each assay, n=10. The data presented were mean ± SD. * *p* < 0.01 vs. group of “Saline”. ^#^ *p* < 0.01 vs. GSK1059615 only.

At last, we studied the anti-tumor activity of GSK1059615 *in vivo*, via a SCC-9 xenograft nude mice model. As demonstrated, *i.p*. daily administration of GSK1059615 at 30 mg/kg significantly inhibited SCC-9 tumor growth in the nude mice (Figure 6A). Estimated tumor growth, expressed as mm^3^ per day [30, 31], was also dramatically inhibited with GSK1059615 administration (Figure 6B). The weight of GSK1059615-treated tumors was also dramatically lighter than those of vehicle control tumors (Figure 6C). Significantly, as shown in Figure 6A-6C, co-administration of cyclosporin A (5 mg/kg, *i.v*., daily) [32], the cyclophilin-D inhibitor, largely attenuated GSK1059615-induced anti-SCC-9 tumor activity. Thus, mPTP and programmed necrosis pathway may also be required for GSK1059615-induced anti-tumor activity *in vivo*. Notably, cyclosporin A alone failed to inhibit SCC-9 tumor growth in the mice (Figure 6A-6C). Importantly, the mice body weight was not significantly different between the groups (Figure 6D), suggesting that these mice were well-tolerated to the tested regimens. Together, GSK1059615 inhibits SCC-9 tumor growth in nude mice, and its anti-tumor activity *in vivo* is compromised with co-administration of cyclosporin A.

## DISCUSSION

mTOR lies in the central position in the PI3K-AKT-mTOR cascade [5–7]. There are two functionally and structurally distinct mTOR complexes, including the mTOR complex 1 (mTORC1, rapamycin-sensitive) and mTOR complex 2 (mTORC2, rapamycin-insensitive) [33]. Both mTOR complexes are important for HNSCC cell progression [33]. We found that GSK1059615 not only simantanuously blocked activation of mTORC1 (indicated by p-S6K1 Thr-389) and mTORC2 (indicated by p-AKT Ser-473), but also inhibited PI3K-AKT activation. This could explain why this compound was more potent in killing HNSCC cells not only than mTOR inhibitors (rapamycin and AZD-2014), but also than the AKT inhibitor (MK-2206 and perifosine). This is also probably why this compound was non-cytotoxic to oral cavity epithelial cells, as these normal cells are with extremely low basal activation of PI3K-AKT-mTOR cascade.

The central function of mitochondrion in regulating intrinsic cell apoptosis has been well-established [34]. Recent studies, interestingly, have proposed the central role of mitochondrion in programming cell necrosis, known as “programmed necrosis” [32, 35–39]. For example, when facing oxidative stresses, p53 will translocate to mitochondrion to trigger mPTP opening, which leads to necrotic, but not apoptotic, cell death [37]. cyclophilin-D deficiency and mPTP blockers were shown to protect cells and experimental animals from a number of necrotic stimuli, including hypoxia, calcium overload, and oxidative stress [37]. Interestingly, multiple anti-cancer agents, *i.e*. cisplatin, doxorubicin, curcumin and icaritin, could also provoke mPTP-dependent programmed necrosis pathway to kill cancer cells [32, 40–42].

In this study, we found that GSK1059615 similarly provoked the programmed necrosis pathway in HNSCC cells, which was evidenced by mitochondria depolarization, ANT-1-cyclophilin-D mitochondrial association and LDH release. Reversely, mPTP blockers or shRNA knockdown cyclophilin-D dramatically alleviated GSK1059615-induced killing of HNSCC cells. In the SCC-9 xenograft tumor nude mice model, co-administration of cyclosporin A significantly attenuated GSK1059615-induced anti-tumor activity. Therefore, targeting PI3K-AKT-mTOR pathway by GSK1059615 possibly provokes programmed necrosis to kill HNSCC cells.

## MATERIALS AND METHODS

### Chemicals and reagents

GSK1059615 was purchased from Adooq Bioscience (Wuxi, China). Gemcitabine and mPTP blockers sanglifehrin A, cyclosporin A and bongkrekic acid as well as wortmannin, LY294002 and perifosine, were provided by Sigma (Shanghai, China). MK-2206, rapamycin and AZD-2014 were purchased from Selleck (Shanghai, China). Antibodies for phospho-PI3K p85 (Tyr458) (#4228), PI3K p85 (#4257), p-AKT (Ser 473, #9271), AKT1 (9272), p-p44/42 MAPK (p-ERK1/2, #9101), ERK1/2 (#9102), p-S6K1 (Thr-389, #9205), mTOR (#2983) and p-mTOR (Ser-2448, #2971) were obtained from Cell Signaling Tech (Shanghai, China). Antibodies for tubulin, adenine nucleotide translocator-1 and cyclophilin-D were obtained from Santa Cruz Biotechnology (Shanghai, China).

### Culture of HNSCC cell lines

SCC-9, SQ20B and A253 cell lines were from Dr. Cui's group [43], and cells were maintained in DMEM medium plus 10% fetal bovine serum (FBS) [43].

### Primary culture human cancer cells and epithelial cells

Four written-informed consent patients with oral cavity carcinoma (OCC) were enrolled, who received no therapy prior to surgery. OCC tissues and the surrounding normal epithelial tissues were obtained at the time of surgery, and were separated very carefully. As described [43], the tissue specimens were washed and incubated with 0.1% collagenase I for digestion. Afterwards, cells were filtered via a 70-μm nylon cell strainer. Primary cells were cultured in complete DMEM/F12 medium with bFGF and EGF [43]. A total of four lines of primary OCC cells (“OCC1-4”) and two lines of oral cavity epithelial cells (“Oepi1-2”) were established. Studies requiring human tissues were reviewed and approved by the Institutional Ethics Committee and Internal Review Committee, and were conducted according to Declaration of Helsinki.

### MTT assay

The routine MTT (Sigma) assay was applied to test cell survival with manufactory's recommendation [44].

### Clonogenicity assay

HNSCC cells (1*10^4^ per 10-cm dish) were trypsinized and suspended in 0.5% agarose-containing complete medium. Ten days following indicated treatment, the survival colonies were stained and counted manually.

### LDH assay

Cell death was tested via medium release of LDH, using a routine two-step LDH assay kit (Takara, Tokyo, Japan) [45]. LDH in the conditional medium was normalized to the total LDH, reflecting cell necrosis percentage [45].

### *In vitro* proliferation assay

Cell proliferation BrdU ELISA assay and [H^3^] thymidine incorporation assay were described in detail in other study [18]. The values were always normalized to the untreated control group.

### ELISA assay of cell apoptosis

The Histone DNA apoptosis ELISA (Roche, Shanghai, China) assay was utilized to quantify cell apoptosis according to the manufacturer's instructions. Detailed protocol can be viewed in other studies [46, 47]. ELISA OD at 450 nm was utilized to quantify cell apoptosis.

### TUNEL assay

The TUNEL *In Situ* Cell Death Detection Kit (Roche, Shanghai, China) was utilized to stain nuclei of apoptotic cells. TUNEL ratio of 200 cells per treatment was recorded.

### Annexin V FACS assay

Cells with treatment were incubated immediately with Annexin V-FITC (5 μg/mL, Invitrogen, Shanghai, China) and the Binding Buffer (Invitrogen), which were subjected to flow cytometry assay of Annexin V.

### Detection of mitochondrial depolarization (ΔΨm)

As described previously [29, 35], JC-10 dye assay (Invitrogen) was performed to reflect mitochondrial membrane potential (MMP) reduction. Briefly, following the treatment, cells were stained with JC-10 (5 μg/mL). Cells were then washed, and JC-10 green fluorescence intensity, indicating MMP reduction (ΔΨm), was tested immediately using a fluorescence microplate reader (Titertek Fluoroscan, Germany).

### shRNA knockdown of cyclophilin-D

The three verified lentiviral cyclophilin-D shRNAs (with non-overlapping sequences) and the nonsense scramble shRNA were gifts from Dr. Xu [29]. The lentiviral shRNA was added directly to cells for 12h. Cells were then subjected to puromycin (5 μg/mL, Sigma) selection for additional 4-5 passages. cyclophilin-D expression in the stable cells was detected by Western blot assay.

### Mitochondrial immunoprecipitation (mito-IP) assay

As described [25], Mitochondria/Cytosol Fractionation Kit (BioVision, Shanghai, China) was utilized to acquire mitochondrial proteins. Six-hundred μg of mitochondrial lysates per treatment were pre-cleared [25]. The lysate supernatant was then rotated overnight with 0.05 μg of designated antibody. Next, the protein A/G PLUS-agarose was added to capture the complex. Pellets were washed, and resuspended in lysis buffer. The immuno-complex was then assayed via Western blot.

### Tumor xenograft assay and IHC staining

Five millions SCC-9 cells per mouse were inoculated *s.c*. into the female nude mice (5-7 week age, 18-20 grams in weight). Within three weeks, established xenograft tumors were established with volumes around 0.1 cm^3^. The mice were then randomized into four groups as mentioned in the text (n=10 per group). Tumor size was measured once every week for a total of 5 weeks, using the modified ellipsoid formula: (π/6) × AB^2^, A represents the longest, and B represents the shortest perpendicular axis of an tumor mass [46]. The animal procedure was approved by the IACUC of authors institutions.

### Statistical analysis

Results were compared by one-way analysis of variance (ANOVA) followed by Turkey's test. Values of *p* < 0.01 were considered as statistically significant.
